# A bleeding colonic ulcer from invasive *Aspergillus* infection in an immunocompromised patient: a case report

**DOI:** 10.1186/1752-1947-8-407

**Published:** 2014-12-05

**Authors:** Jorge Bizet, Chad J Cooper, Marc J Zuckerman, Alireza Torabi, Antonio Mendoza-Ladd

**Affiliations:** Department of Internal Medicine, Texas Tech University Health Sciences Center, 4800 Alberta Ave, El Paso, TX 79905 USA

**Keywords:** Invasive *Aspergillus*, Aspergillosis, Gastrointestinal bleeding, Immunosuppression

## Abstract

**Introduction:**

Invasive *Aspergillus* commonly involves the lungs, but can also affect other organs such as the skin, adrenal glands, central nervous system, liver, spleen and the gastrointestinal tract. Gastrointestinal aspergillosis is rare and is most often discovered in immunocompromised patients. There is only one other case report to our knowledge that describes the diagnosis being discovered on histopathological analysis of endoscopic biopsies of necrotic ulcers.

**Case presentation:**

A 36-year-old Hispanic woman presented with septic shock secondary to extensive Fournier gangrene that required multiple surgical debridement of the perineal and retroperitoneal area. Her vital signs on admission were a temperature of 39.4°C and blood pressure of 85/56mmHg, pulse rate of 108/min and respiratory rate of 25. An examination of the perineum/genital area revealed bilateral gluteal and perilabial edema, erythema and focal areas of necrotic tissue with purulent discharge. Other surgeries included small bowel resections with ileoileal anastomosis that later developed an anastomotic leak that required and diverting end ileostomy. Eleven weeks after admission, our patient developed hematochezia from the colostomy associated with a decrease in hemoglobin and hematocrit to 6.4g/dL and 20.2% respectively. Colonoscopy through the ostomy revealed blood throughout the colon and a 3cm necrotic ulcer with an adherent clot in the transverse colon. Biopsies were taken from the edge of the ulcer. Histopathological analysis of the specimen with Grocott’s methenamine silver stain revealed septated hyphae with the 45-degree-angle branching that is morphologically consistent with *Aspergillus* species. Our patient was treated with intravenous voriconazole for 30 days with a prolonged hospitalization but no recurrent bleeding.

**Conclusions:**

Gastrointestinal aspergillosis is an unusual presentation of invasive *Aspergillus* associated with a high mortality rate. Characteristic features of gastrointestinal aspergillosis include invasion of the mesenteric arteries, intravascular thrombosis and subsequent tissue ischemia. Clinical manifestations of invasive *Aspergillus* of the gastrointestinal tract can include fever, abdominal pain, ileus, peritonitis, bloody diarrhea or hematochezia. In an autopsy series of patients with invasive *Aspergillus*, 37 of 107 patients had *Aspergillus* involvement of the gastrointestinal system; the most common pathological findings included ulcers and abscesses. Although rare, invasive aspergillosis may present with gastrointestinal bleeding associated with necrotic ulcers on endoscopic examination.

## Introduction

*Aspergillus* species are ubiquitous saprophytic spore-producing fungi commonly found in the environment. Out of its approximately 200 species, *Aspergillus fumigatus* is responsible for the vast majority of cases of human invasive aspergillosis, a potentially fatal illness that affects the lungs and other organs of immunocompromised patients. Invasive aspergillosis is associated with a 50 to 60% mortality rate [1, 2]. Other potential species include *Aspergillus niger, Aspergillus flavus* and *Aspergillus terreus* [3]. Human disease usually begins with inhalation or ingestion of its airborne conidia. These conidia are deposited in the sinuses, bronchioles and alveoli. In healthy individuals, the spores that are not removed through mucociliary clearance are eliminated through phagocytosis and a pro-inflammatory response by alveolar macrophages [4]. Occasionally, conidia may evade destruction by the alveolar macrophages. In these cases, neutrophils kill any remaining pathogens. If the conidia evade the host defenses, they reach the bloodstream and invade the endothelial cell lining of the blood vessels, disseminating to various organs through a hematogenous route. Therefore, if a dysfunction exists in the host’s cellular immune system, the risk of developing invasive aspergillosis (IA) increases. Neutropenia and corticosteroid use are two of the most common conditions associated with this scenario.

The lungs are the most common organ affected by IA, but given its hematogenous spread, other sites, such as the gastrointestinal tract may also be involved. *Aspergillus* spores usually do not survive on the mucosal surfaces of the gastrointestinal tract. However, it has been proposed that any mechanism that causes disruption of the gastrointestinal mucosa can predispose the bowel to IA.

## Case presentation

A 36-year-old Hispanic woman presented with severe perineal pain radiating to the right gluteal area for one week. Her associated symptoms included fever, chills, nausea, fatigue and shortness of breath. She denied any vaginal discharge, dysuria, changes in stool color, bloody stools, vomiting or diarrhea. No past medical problems were noted. Her social history was relevant for a long-standing history of alcohol use and smoking. Her vital signs on admission revealed a temperature of 39.4°C, blood pressure of 85/56mmHg, heart rate of 109/min and respiratory rate of 25/min. A physical examination revealed rhonchi in both lung fields, no heart murmurs, and diffuse abdominal tenderness without guarding or rebound signs. An examination of the perineum/genital area revealed bilateral gluteal and perilabial edema and erythema, associated with increased local temperature and focal areas of necrotic tissue with purulent discharge.

Initial laboratory results upon admission were a white blood cell count of 6.09 × 103uL, hemoglobin of 17.9g/dL, hematocrit of 51.8%, platelet count of 53 × 10^3^/uL, blood urea nitrogen of 32mg/dL, creatinine of 1.77mg/Dl and serum glucose of 196mg/dL. A computed tomography (CT) scan of the abdomen and pelvis revealed extensive necrotizing fasciitis at the right gluteal region extending into the pelvis, retroperitoneal space and the anterior abdominal wall. The posterior and medial compartments of the right thigh were also involved. Our patient was admitted to the surgical intensive care unit with a diagnosis of septic shock secondary to Fournier’s gangrene. The first surgery was surgical debridement of the right perineum, perirectal ischioanal and gluteus region. Additional surgical procedures included exploratory laparotomy, debridement of retroperitoneum and bilateral anterior abdominal wall and drainage of pelvic abscesses. She had multiple explorations of the abdomen and perineal area with debridement of necrotic tissue. She later developed a small bowel perforation of the ileum resulting in fecal peritonitis. She therefore had a small bowel resection with ileoileal anastomosis. One week later, she developed an anastomotic leak that required resection of the anastomosis and creation of an end ileostomy. Our patient subsequently developed pancreatic necrosis with abscess formation requiring exploratory laparotomy for drainage of the abscess and debridement of pancreatic necrosis. Eight weeks after admission, she developed dry gangrene and necrosis of all limbs ensued requiring amputation of all the fingers of her right hand, left wrist disarticulation and open disarticulation of all her toes.

**Figure 1:**
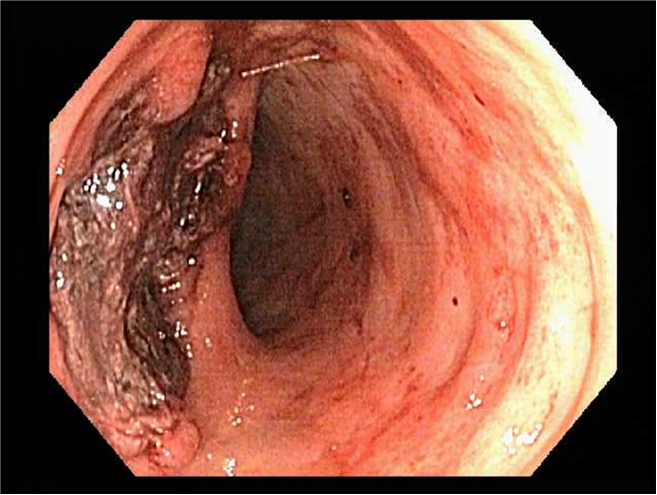
**A 3cm necrotic ulcer with an adherent blood clot in the transverse colon.**

**Figure 2:**
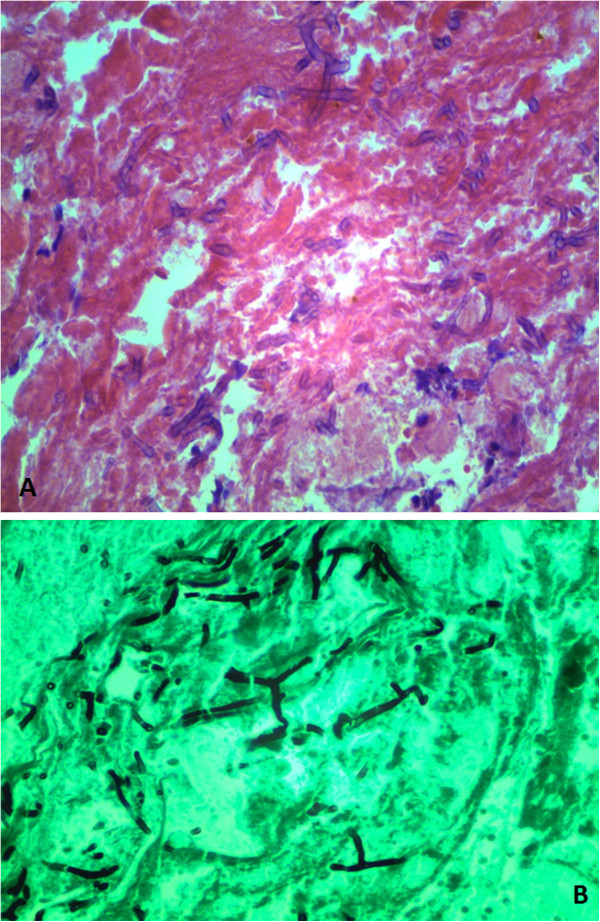
**(A) Hematoxylin and eosin stain showing fungi micro-organism amid necrotic debris, blood and fibrin. (B)** Grocott's methenamine silver stain highlighting septated hyphae with 45-degree-angle branching, morphologically consistent with *Aspergillus* species.

Eleven weeks after admission, our patient developed hematochezia from the colostomy associated with a decrease in hemoglobin from 11.4g/dL to 6.4g/dL. Our patient was initially stabilized with continuous intravenous pantoprazole infusion and received four units of packed red blood cells. Colonoscopy through the ostomy revealed blood throughout the colon and a 3cm necrotic ulcer with an adherent clot in the transverse colon (Figure 1A). Multiple biopsies were taken from the edge of the colonic ulcer. Pathological analysis of the specimen (Figure 2A and 2B) with hematoxylin and eosin (H&E) and Grocott’s methenamine silver (GMS) stain revealed septated hyphae with 45-degree-angle branching morphologically consistent with *Aspergillus* species. Our patient was then immediately treated with intravenous voriconazole, 360mg every 12 hours for a total of 30 days. One month after the initial colonoscopy, melena was seen in the ileostomy bag. An upper endoscopy revealed superficial non-bleeding erosions in the esophagus and gastric body. A pathological analysis revealed chronic gastritis and no fungal organisms. No further bleeding episodes were noted. She continued to have a complicated hospital course associated with pleural effusions, sacral decubitus ulcers, and intramuscular abscesses of her thighs and pelvic area. At eight-month follow-up, she was still in the hospital undergoing further care but had no recurrent gastrointestinal bleeding.

## Discussion

Although the lung is the most commonly affected site in IA, the involvement of other sites, such as the central nervous system, paranasal sinuses, heart, bones, joints, eyes, skin, kidneys, and gastrointestinal tract have been reported [5]. The gastrointestinal tract is the second most common site of invasive aspergillosis, and the small intestine is its most affected organ [5, 6]. Gastrointestinal aspergillosis typically occurs in the settings of disseminated infection and its frequency in this scenario is approximately 17% based on several autopsy studies [5, 7, 8]. Characteristic features of gastrointestinal aspergillosis include invasion of the mesenteric arteries, intravascular thrombosis and subsequent tissue ischemia. This ischemic lesion leads to infarction and even perforation of the intestine. IA of the small bowel has a typical macroscopic appearance of a thickened and purplish wall with islands of necrotic tissue [8–10].

Clinical manifestations of IA of the gastrointestinal tract can include fever, abdominal pain, ileus, peritonitis, bloody diarrhea or hematochezia. Radiographic findings of diffuse small bowel distention and wall thickening may aid in the diagnosis [11]. However, there are no specific radiologic findings that will immediately suggest IA of the gastrointestinal tract. Common surgical or endoscopic findings in previously reported cases include ulcerative or necrotic lesions.

Most of the literature on gastrointestinal IA comes from case reports or case series as summarized in Table 1. Eggiman *et al.* reported on two cases and reviewed eight previously published cases in the literature. Nine patients had a hematologic malignancy with eight of them receiving cytarabine [3]. This chemotherapeutic agent has a cytotoxic effect on the colonic mucosa that disrupts the normal mucosal barriers and allows invasion by *Aspergillus* [12]. High doses of cytarabine-induced mucositis can lead to ulceration and a favorable environment for colonization by *Aspergillus* spores [13]. Cohen *et al.* reported a neutropenic patient with acute myelogenous leukemia treated with cytarabine and idarubicin who presented with fever and bloody diarrhea secondary to bowel infarction from IA [9]. However, not all patients had received chemotherapy, as described in a case by Choi *et al*. In this case, isolated IA of the colon was diagnosed in a patient with colon cancer who was not receiving chemotherapy [14].

**Table 1:** **Case reports of invasive** ***Aspergillus*** **in the gastrointestinal tract**

Authors	Underlying diagnosis	Previous chemotherapy	Clinical presentation	Portion of the gastrointestinal tract involved	Intervention (surgery/endoscopy)	Antifungal treatment
**Karaman** ***et al*** **.** [5]	Arthrogryposis multiplex congenita	None	Abdominal pain and vomiting	Stomach	Surgery	None.
**Eggiman** ***et al*** **.** [3]	Acute myeloid leukemia (2 cases)	Cytarabine, idarubicin	Fever, bloody diarrhea bleeding, ileus or peritonitis	Small intestine	Surgery	Amphotericin B
**Cohen** ***et al*** **.** [9]	Acute myeloid leukaemia	Cytarabine, daunorubicin	Fever, watery diarrhea, peritonitis	Small intestine	Surgery	None. Patient died.
**Trésallet** ***et al*** **.** [11]	Lymphoma	Cytarabine, etoposide	Fever, peritonitis	Small intestine	Surgery	Voriconazole
**Chaudhary** ***et al*** **.** [6]	Breast carcinoma	Cyclophosphamide, adriamycin, 5**-**fluorouracil	Fever, constipation, abdominal pain	Small intestine	None	Amphotericin B
**Jayshree** ***et al*** **.** [10]	Acute promyelocytic leukemia	Daunomycin, cytosine arabinoside	Fever and diarrhea	Colon	Surgery	Amphotericin B
**Choi** ***et al*** **.** [14]	Colon cancer	None	Hematochezia	Colon	Endoscopy	Amphotericin B
**Bizet** ***et al*** **. (Current case)**	Fournier’s gangrene	None	Hematochezia through colostomy	Colon	Endoscopy	Voriconazole

Gastrointestinal IA may be more common than originally thought. In a series of 107 autopsy reports of patients with IA [1], 37 had gastrointestinal IA (16 with upper tract involvement, nine with lower tract involvement, seven hepatic IA and five pancreatic IA). The two most common pathological findings in these patients included ulcers and abscesses. Of the 16 patients with upper gastrointestinal tract involvement, eight were asymptomatic, five had upper abdominal pain and three had massive melena. Among the nine with lower gastrointestinal tract involvement, four were asymptomatic, two had hematochezia, one had a mass in the colon, one had peritonitis and one had ileus.

The definitive diagnosis of IA requires histopathological evidence of infection such as direct microscopy of fluid or tissue specimens showing the characteristic angular dichotomously branching septated hyphae and confirmatory culture results [15]. However, if cultures and microscopy are negative, then the diagnosis relies on the surrogate non-culture-based method in patients with compatible clinical manifestations and risk factors [14]. These non-culture-based methods include galactomannan or (1,3) ß-D-glucan assay. Galactomannan and (1,3) ß-D-glucan are components of the cell wall of the *Aspergillus* mold released during the growth of hyphae [14]. Galactomannan antigen is detected in body fluids through an enzyme-linked immunosorbent assay (ELISA). However, this assay is not specific for the type of *Aspergillus* species. The initial therapy for IA includes voriconazole, but amphotericin B can be used if the patient is unable to tolerate voriconazole [16, 17]. Surgical treatment of gastrointestinal aspergillosis is required for complications, such as perforation, obstruction, bleeding and infarction.

## Conclusions

Gastrointestinal aspergillosis is an unusual presentation of invasive aspergillosis and is associated with a high mortality rate. Due to its low incidence, IA is not routinely considered as a differential diagnosis of lower gastrointestinal bleeding. However, in the setting of an immunocompromised patient secondary to sepsis with gastrointestinal bleeding found to have necrotic ulcers on endoscopic examination, IA should be included in the differential diagnosis.

## Consent

Written informed consent was obtained from the patient for publication of this case report and any accompanying images. A copy of the written consent is available for review by the Editor-in-Chief of this journal.

## Abbreviations

CT: computed tomography
ELISA: enzyme-linked immunosorbent assay
GMS: Grocott’s methenamine silver
H&E: hematoxylin and eosin
IA: invasive *Aspergillus* .
